# Realizing Plain Optimization of the Thermoelectric Properties in BiCuSeO Oxide via Self-Substitution-Induced Lattice Dislocations

**DOI:** 10.34133/research.0123

**Published:** 2023-04-18

**Authors:** Rui Xu, Zhiwei Chen, Qizhu Li, Xiaoyu Yang, Han Wan, Mengruizhe Kong, Wei Bai, Nengyuan Zhu, Ruohan Wang, Jiming Song, Zhou Li, Chong Xiao, Binghui Ge

**Affiliations:** ^1^ Information Materials and Intelligent Sensing Laboratory of Anhui Province, Key Laboratory of Structure and Functional Regulation of Hybrid Materials of Ministry of Education, Institutes of Physical Science and Information Technology and School of Materials Science and Engineering, Anhui University, Hefei 230601, China.; ^2^ Institute of Energy, Hefei Comprehensive National Science Center, Hefei 230031, China.; ^3^ School of Materials Science and Engineering, Tongji University, Shanghai 201804, China.; ^4^ Hefei National Laboratory for Physical Sciences at the Microscale, CAS Center for Excellence in Nanoscience, University of Science and Technology of China, Hefei 230026, China.

## Abstract

Seeking new strategies to tune the intrinsic defect and optimize the thermoelectric performance via no or less use of external doped elements (i.e., plain optimization) is an important method to realize the sustainable development of thermoelectric materials. Meanwhile, creating dislocation defects in oxide systems is quite challenging because the rigid and stiff ionic/covalent bonds can hardly tolerate the large strain energy associated with dislocations. Herein, taking BiCuSeO oxide as an example, the present work reports a successful construction of dense lattice dislocations in BiCuSeO by self-doping of Se at the O site (i.e., Se_O_ self-substitution), and achieves plain optimization of the thermoelectric properties with only external Pb doping. Owing to the self-substitution-induced large lattice distortion and the potential reinforcement effect by Pb doping, high-density (about 3.0 × 10^14^ m^−2^) dislocations form in the grains, which enhances the scattering strength of mid-frequency phonon and results in a substantial low lattice thermal conductivity of 0.38 W m^−1^ K^−1^ at 823 K in Pb-doped BiCuSeO. Meanwhile, Pb_Bi_ doping and Cu vacancy markedly improve the electrical conductivity while maintaining a competitively high Seebeck coefficient, thereby contributing to a highest power factor of 942 μW m^−1^ K^−2^. Finally, a remarkably enhanced *zT* value of 1.32 is obtained at 823 K in Bi_0.94_Pb_0.06_Cu_0.97_Se_1.05_O_0.95_ with almost compositional plainification. The high-density dislocation structure reported in this work will also provide a good inspiration for the design and construction of dislocations in other oxide systems.

## Introduction

Facing the problems of low efficiency and high carbon emission in current energy utilization, and targeting the “carbon peaking and carbon neutrality” goals, the energy sector has put forward more urgent needs for green and efficient energy conversion materials and technologies [1,2]. Thermoelectric materials, based on the migration of carriers under temperature gradient or electric current, enable direct and reversible conversion between heat and electricity, which will not produce any pollutant emission during the operation and theoretically has maximum energy conversion efficiency equal to the Carnot efficiency [3,4]. Therefore, they represent a kind of novel green and efficient energy materials for power generation and cooling and have become a research hotspot in the field of new energy materials for low-carbon energy conversion [5,6]. Meanwhile, in addition to greenness and efficiency aspects, the retrievability is another practical facet that requires scientific attention during the full-life cycle and material sustainability research [7,8], which is particularly important for thermoelectric materials and devices considering their bulk character with larger consumption of raw materials. For example, in the superalloys used for aero-engines, there are more than 10 alloy elements, including rare and precious metals such as rhenium (Re) and platinum (Pt) [9], which leads to a high cost and recovery difficulties, and also makes the improvement of the material performance being overdependent on the external doped alloy elements. In order to facilitate recovery and reduce costs, low alloying or, more generally, reducing the use of external doping elements in material modification is particularly noteworthy. In recent years, to promote material recycling and improve sustainability, Lu and et al. [9,10] proposed a concept of “material plainification” in metal material research, which emphasizes the importance of intrinsic microstructure for performance modulation and advocates low or even no alloy addition to maintain the high performance of materials. This novel concept sheds light on the sustainable research of thermoelectric materials and inspires us that high thermoelectric performance can also be achieved under the condition of no or less use of external doped elements (i.e., plain optimization) by tuning intrinsic microstructures in materials [11].

The performances of thermoelectric materials are normally assessed by the dimensionless figure of merit, denoted as *zT*. The larger the *zT* value, the higher the thermoelectric conversion efficiency. Its expression is *zT* = *S*^2^*σ/κ*, where *T*, *S*, *σ*, and *κ* are the absolute temperature, Seebeck coefficient, electrical conductivity, and thermal conductivity, respectively [12]. Meanwhile, *κ* is mainly composed of electronic (*κ*_e_) and lattice (*κ*_l_) thermal conductivity, i.e., *κ* = *κ*_e_ + *κ*_l_. Since *S* and *σ* have a strong inverse coupling relationship, and *κ*_e_ is proportional to *σ* according to the Wiedemann–Franz law *κ*_e_ *= LσT* (*L* is the Lorentz constant) [13–15], it is thus quite difficult to control these thermoelectric transport parameters separately and gain a desired high *zT* value. Basically, the *zT* improvement of a thermoelectric material relies on improving the power factor (*PF* = *S*^2^*σ*) and decreasing the *κ*_l_ [16–19]. The ways to achieve the former goal includes the defect-induced optimizations of carrier concentration [20–22], mobility [23,24], band modulation [25,26], etc. On the other hand, defect manipulations by atom doping, compositing, and nanostructuring are usually used to suppress the phonon diffusion and achieve lower *κ*_l_. Combining the demands for high-performance and sustainability, decoupling the thermoelectric parameters by well-designed intrinsic defect modulations and improving the *zT* performance with compositional “plainification” should be a scientific frontier and worthy of research in the thermoelectric community.

To achieve the above scenario, we should turn our sights to the intrinsic defects in thermoelectric material. In fact, intrinsic point defects, such as vacancy [27–30], interstitial [15,31], and self-substitution atoms, have demonstrated the comparable or even better capabilities in improving the thermoelectric performance. Moreover, high-dimensional defects, such as nanoprecipitates, twins/phases/grain boundaries, and lattice dislocations, have also cut a striking figure in thermoelectric research via blocking heat propagation and achieving a satisfactory low *κ*_l_ [31–33]. Among them, lattice dislocation is particularly prominent due to its great contribution to the mid-frequency-range phonon blocking and the difficulty of generation in non-alloy compounds [32,33]. This is due to the broad distribution of phonons wherein the mid-frequency phonons have the dominant contribution to the *κ*_l_. Therefore, introducing dislocations can help to reduce the *κ*_l_ to the greatest extent. On the other hand, for metal and alloy systems, high-density dislocations can be easily introduced through plastic deformation and vacancy engineering [34,35]. For instance, Rogl et al. reported high-density dislocations through high-pressure torsional plastic deformation treatment of CoSb_3_ skutterudite [36]. Our previous work also demonstrated that cation vacancies in the Pb_1−*x*_Sb_2*x*/3_Se system collapsed to form dense dislocations in the grains [37]. However, creating dislocations in non-alloy compounds, especially oxide systems, is quite uncommon, mainly because the rigid and stiff ionic/covalent bonds in oxides can hardly withstand the enormous strain energy associated with dislocations [38]. Therefore, it is challenging but very important to explore the lattice dislocations in oxide thermoelectric systems, which will help to realize plain optimization and illuminate the construction of dislocations.

**Fig. 1.**
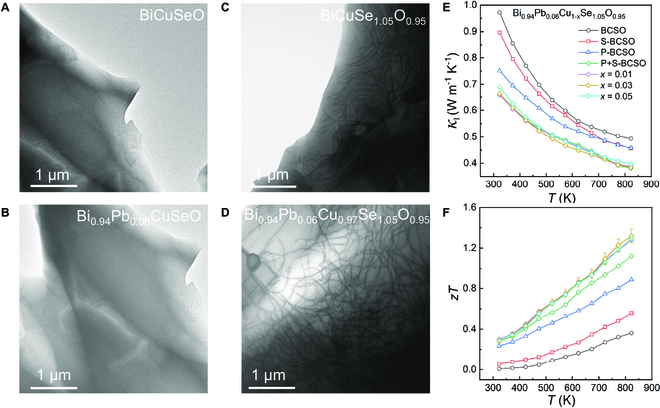
(A to D) STEM-ABF images of pristine BiCuSeO (A), Bi_0.94_Pb_0.06_CuSeO (B), BiCuSe_1.05_O_0.95_ (C), and Bi_0.94_Pb_0.06_Cu_0.97_Se_1.05_O_0.95_ (D) samples. Uniformly distributed dense dislocation lines are observed in Se_O_ self-substitution samples, which do not exist in pristine and Pb-single-doped BiCuSeO. (E) The temperature-dependent lattice thermal conductivity and (F) *zT* value of BiCuSeO, BiCuSe_1.05_O_0.95_, Bi_0.94_Pb_0.06_CuSeO, and Bi_0.94_Pb_0.06_Cu_1−*x*_Se_1.05_O_0.95_ (*x* = 0, 0.01, 0.03, and 0.05) samples.

As a typical thermoelectric oxide, BiCuSeO has multiple component elements and special quasi-superlattice structure with [Bi_2_O_2_]^2+^ and [Cu_2_Se_2_]^2−^ sublayers alternately stacking along the *c* axis [39–41], which endows it with rich intrinsic defect configurations, thereby providing a good platform for us to conduct the intrinsic defect research and particularly explore the dislocation effect. Herein, this work successfully constructs dense in-grain dislocations in BiCuSeO by self-doping of Se at the O site (i.e., Se_O_ self-substitution) and achieves plain optimization of the thermoelectric properties with only external Pb doping. Firstly, large lattice distortion is induced by Se_O_ self-substitution, which promotes the formation of high-density dislocations that do not exist in pristine and Pb_Bi_-single-doped BiCuSeO (Fig. 1A to D). This complements weakness in the mid-frequency phonon scattering and gives rise to a substantial low *κ*_l_ of 0.38 W m^−1^ K^−1^ at 823 K in the Bi_0.94_Pb_0.06_Cu_0.97_Se_1.05_O_0.95_ sample (Fig. 1E). Meanwhile, Pb_Bi_ doping and Cu vacancy markedly improve *σ* while maintaining a competitively high *S*, thereby contributing to a high *PF* of 942 μW m^−1^ K^−2^. As a result, the final thermoelectric performance is remarkably enhanced in Bi_0.94_Pb_0.06_Cu_1−*x*_Se_1.05_O_0.95_, and the highest *zT* value of 1.32 is obtained in Bi_0.94_Pb_0.06_Cu_0.97_Se_1.05_O_0.95_ at 823 K (Fig. 1F). The much-improved *zT* performance is obtained by tailoring the intrinsic defects with almost compositional plainification (with only external Pb dopant), embodying the concept of “plain optimization”. Meanwhile, the high-density dislocation structure reported in this work will also provide a good inspiration for the design and construction of dislocations in other oxide systems.

## Results and Discussion

Serial BiCuSeO-based thermoelectric samples, including pristine BiCuSeO (BCSO), Pb_Bi_-single-doped Bi_0.94_Pb_0.06_CuSeO (P-BCSO), Se_O_-single-doped BiCuSe_1.05_O_0.95_ (S-BCSO), and co-doped Bi_0.94_Pb_0.06_Cu_1−*x*_Se_1.05_O_0.95_ (P+S-BC_1−*x*_SO, *x* = 0, 0.01, 0.03, 0.05), were prepared by vacuum solid-state reaction followed by the hot-pressing process. As mentioned above, Fig. 1A to D show the low-magnification scanning transmission electron microscopy-annular bright field (STEM-ABF) images of samples BCSO, P-BCSO, S-BCSO, and P+S-BC_0.97_SO, respectively. Compared with the pristine and Pb-single-doped BiCuSeO (Fig. 1A and B), we can clearly find that the S-BCSO and P+S-BC_0.97_SO samples contain a large number of uniformly distributed dislocation lines (Fig. 1C and D). The existence of these dislocations can also be reflected in TEM mode. Figure S1 shows that (see in the Supplementary Materials) there are many bumps and scars in the matrix of S-BCSO and P+S-BC_0.97_SO, while it is smooth and flat for that of BCSO and P-BCSO samples, further indicating the existence of dislocations. The *κ*_l_ of S-BCSO and P+S-BC_0.97_SO decreases greatly compared with that of BCSO and P-BCSO (Fig. 1E), which is consistent with the expectation since dislocations can effectively scatter mid-frequency phonons. As shown in Fig. 1E, the minimum *κ*_l_ reaches 0.38 W m^−1^ K^−1^ at 823 K. Finally, the thermoelectric *zT* performance of the P+S-BC_0.97_SO sample was greatly improved, peaking at 1.32 at 823 K (Fig. 1F). This *zT* value is also highly competitive with the most advanced oxide thermoelectric systems at the same temperature, as shown in Fig. S2.

**Fig. 2.**
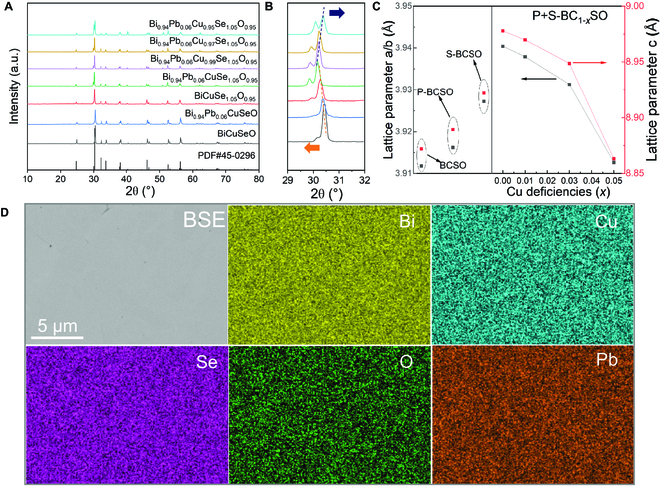
(A) XRD patterns of BiCuSeO, BiCuSe_1.05_O_0.95_, Bi_0.94_Pb_0.06_CuSeO, and Bi_0.94_Pb_0.06_Cu_1−*x*_Se_1.05_O_0.95_ (*x* = 0, 0.01, 0.03, and 0.05) samples. (B) Enlarged XRD patterns of region 29° to 32°. (C) The derived lattice parameters. (D) BSE and elemental mapping images of sample Bi_0.94_Pb_0.06_Cu_0.97_Se_1.05_O_0.95_, showing that all elements in the region are uniformly distributed, without enrichment or precipitation.

Beside the lattice dislocations observed here, other inherent defects such as point defects, grain boundaries, and secondary precipitation contribute to the reduction of *κ*_l_. The phase analysis provides the defect information, which was characterized by x-ray diffraction (XRD), backscattered electron (BSE), and energy-dispersive spectrometer (EDS) elemental mapping with the results shown in Fig. 2. The XRD pattern of the sample (Fig. 2A) is consistent with the standard diffraction pattern of tetragonal BCSO (PDF No. 45-0296), indicating that no impurity phases were revealed within the detection limit of the instrument and the doped elements enter the BCSO crystal lattice. After detailed analysis of the diffraction pattern at 29° to 32° as shown in Fig. 2B, we find that when Pb is doped at the Bi site, the main peak of the blue line shifts to the lower angle because of the larger ion radius of Pb^2+^ than that of the Bi^3+^, and it is analogous to the substitution of O by Se. When Pb and Se are doped at the same time, the offset reaches the maximum at this time, as shown by the green line in Fig. 2B. By contrast, with the increase of Cu vacancies, the main peak of P+S-BC_1−*x*_SO gradually shifted to the higher angle. This change can also be clearly seen from the plots of derived lattice parameters in Fig. 2C. Moreover, the BSE image and corresponding elemental mapping of the P+S-BC_0.97_SO block after hot pressing (Fig. S3 and Fig. 2D) confirm again that there is no obvious secondary phase in the sample and all elements in the region are uniformly distributed, without enrichment or precipitation.

It is also worth noting that, in addition to the shift of peaks, the main diffraction peaks in Fig. 2B show obvious widening in S-BCSO and P+S-BC_1−*x*_SO samples, which indicates the existence of large lattice distortion accompanying with the observed dense dislocations. On the other hand, since the widening of the diffraction peak is strictly related to the dislocation density and grain size, through the modified Williamson–Hall analysis of the XRD peak, we analyzed the ΔK-K^2^C plots for samples S-BCSO and P+S-BCSO (Fig. S4), in which the slope of the plots reveals the dislocation density, and obtained dislocation density of the sample P+S-BC_0.97_SO macroscopically about 3.0 × 10^14^ m^−2^ and the sample S-BCSO macroscopically about 4.0 × 10^13^ m^−2^. The details of the calculation process can be found in previous works [37,42–45]. Because the presence of the dislocation can be effective in scattering mid-frequency phonons, the *κ*_l_ of S-BCSO is lower than that of the pure BCSO sample, and the *κ*_l_ of P+S-BCSO is also lower than that of the P-BCSO sample (Fig. 1E). The formation mechanism of dislocation is thought to be attributed to the strong lattice distortion caused by self-doping of Se at the O site (i.e., Se_O_ self-substitution), which can provide extra energy for dislocation generation [33]. It can be seen from the XRD pattern in Fig. 2B that the main diffraction peak of the S-BCSO sample is more shifted to the lower angle than that of the pure BCSO and P-BCSO, indicating that there is a large lattice distortion in the sample that can supply the energy for dislocation generation. Compared with S-BCSO, the main diffraction peak of P+S-BCSO continues to shift to a further lower angle, indicating a larger lattice distortion compared to the S-BCSO and resulting in a higher dislocation density (4.0 × 10^13^ m^−2^ versus 3.0 × 10^14^ m^−2^), which is consistent with the relative larger reduction of *κ*_l_ in the P+S-BCSO sample (Fig. 1E). Present results also imply that Pb_Bi_ doping may promote the dislocation formation on the basis of Se_O_ self-substitution, but the underlying mechanism is unclear now and worth further study in future work.

**Fig. 3.**
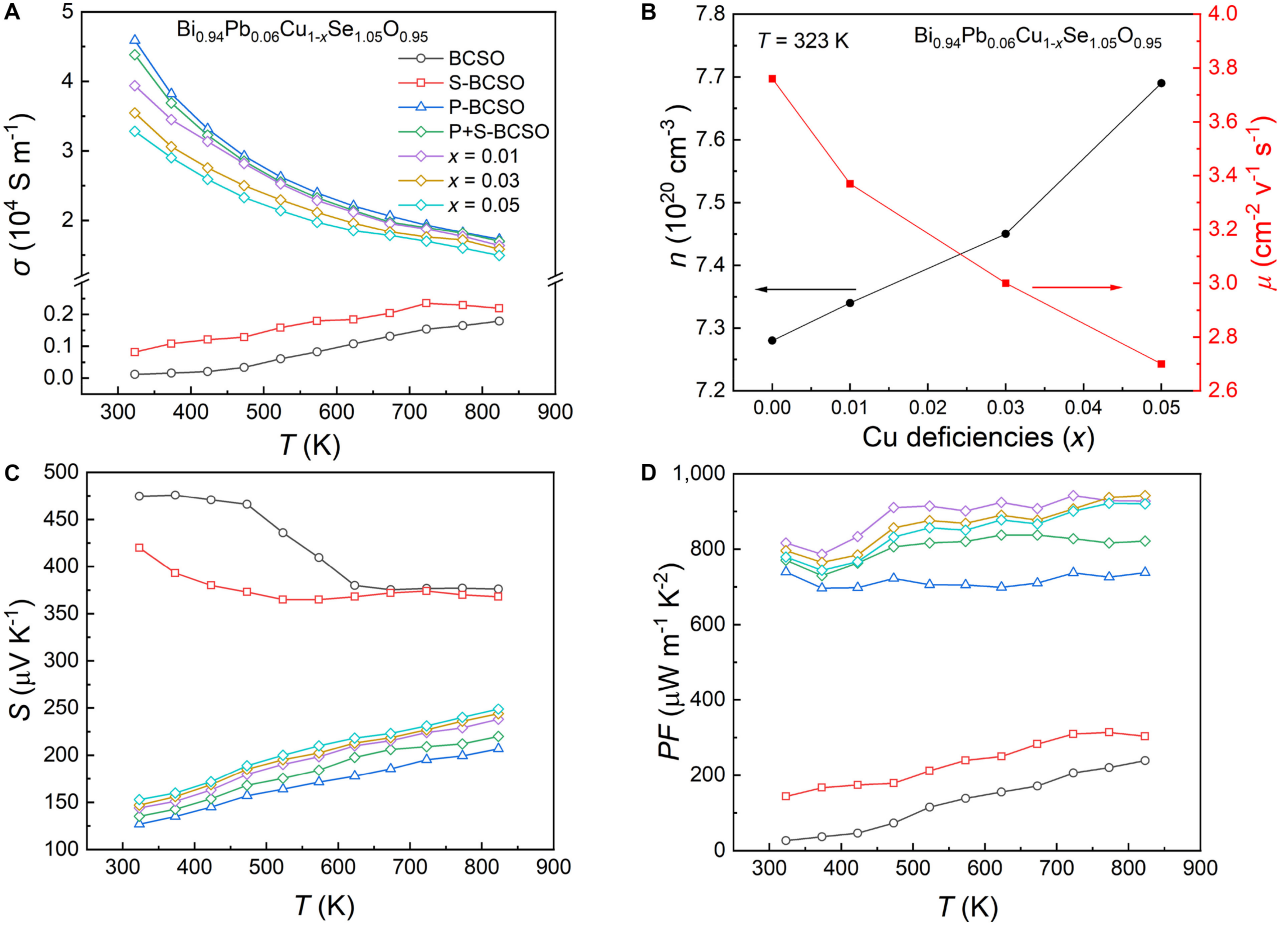
Electrical transport properties. (A to D) Temperature-dependent electrical conductivity (A), carrier concentration and mobility at 323 K (B), temperature-dependent Seebeck coefficient (C), and power factor (D) of the BiCuSeO, BiCuSe_1.05_O_0.95_, Bi_0.94_Pb_0.06_CuSeO, and Bi_0.94_Pb_0.06_Cu_1−*x*_Se_1.05_O_0.95_ (*x* = 0, 0.01, 0.03, and 0.05) samples.

The results of the electrical transport properties of the samples in the temperature range 323 to 823 K are shown in Fig. 3. Figure 3A shows the variation of *σ* with temperature for the BCSO, S-BCSO, P-BCSO, and P+S-BC_1−*x*_SO (*x* = 0, 0.01, 0.03, and 0.05) samples. As shown in Fig. 3A, BCSO and S-BCSO samples exhibit non-degenerate semiconductor behavior as the *σ* increases with temperature throughout the temperature interval, while the remaining samples exhibit metallic behavior as their electrical conductivities decrease with increasing temperature. At a temperature of 323 K, pristine BCSO has the lowest *σ* of 117 S m^−1^ among all samples, which is because of the low intrinsic carrier concentration (*n*) of BCSO [28]. When the element Se substitutes the O position, the *σ* slightly increases from 117 S m^−1^ to 915 S m^−1^, and hugely increases after Pb_Bi_ doping, reaching 45,897 S m^−1^ (blue line in Fig. 3A). To explore the underlying reasons for the increase, the *n* and mobility (*μ*) of the samples at 323 K were obtained using the Hall coefficient test, as shown in Fig. S5A. When element Se equivalently substitutes O by 5% (i.e., sample S-BCSO), *n* is slightly enhanced. This may be because of the weaker Bi–Se bond strength compared to the original Bi–O bond, which is in favor of the generation of acceptor-type V_Bi_ defects and contributes modest amount of hole carriers. At the same time, as can be seen from Fig. S6, the S-BCSO sample also becomes more compact with the Se_O_ substitution, showing larger grain size and lower grain boundary density than BCSO, which results in much lower grain boundary scattering and thus higher *μ* (see Fig. S5A). The combined effect of the 2 factors finally results in a higher *σ* of S-BCSO [46,47]. Meanwhile, Pb doping at the Bi site (i.e., sample P-BCSO) provides more hole carriers without a marked decrease in *μ*, leading to a substantial increase in *σ*.

Compared with P-BCSO, the *σ* of Se_O_ and Pb_Bi_ co-doped P+S-BCSO basically remained unchanged, but reduced gradually with the increase of Cu vacancies (Fig. 3A). In principle, with the increase of Cu vacancies, *n* should be further increased and so does *σ*, due to the acceptor character of V_Cu_ [28,29]. To elucidate this anomaly, we also performed the Hall test at 323 K and obtained the *n* and *μ* data of P+S-BC_1−*x*_SO (*x* = 0, 0.01, 0.03, and 0.05) samples. As shown in Fig. 3B, with the increase of Cu vacancies, *n* gradually increases as expected, but *μ* decreases greatly. It can be deduced from the formula *σ = neμ* that the reduction in *σ* is mainly caused by the decrease in *μ*, which may be due to the following reasons: (a) On the one hand, the existence of Cu vacancies easily scatters the carriers. With the increase of Cu vacancies, the scattering intensity of carriers becomes stronger and stronger, which greatly damaged the transport of carriers [48]. (b) On the other hand, as *n* increases, the mutual scattering between the carriers also becomes stronger, which also has a considerable effect on *μ*. Combined with the above factor, the *σ* of the P+S-BC_1−*x*_SO sample decreases with the increase of Cu vacancies.

Figure 3C shows the data of the temperature-dependent *S* of the samples. Obviously, since *S* is inversely proportional to *n*, the *S* of the doped samples is lower than that of the pure BCSO sample due to the increase of *n*. However, worthy of note is that the *S* of the P+S-BC_1−*x*_SO samples shows a reverse increasing trend though *n* continues to increase compared to that of P-BCSO. To explore the origin of enhancement in *S*, the corresponding Pisarenko curves were plotted with different density of state effective masses (m^*^) based on the parabolic single band structure and shown in Fig. S5b. It could be clearly found that the data points of “*S* versus *n*” for P+S-BC_1−*x*_SO fall on the Pisarenko curves with higher m^*^. Specifically, as shown in Fig. S5b, the data point for P-BCSO locates on the blue curve, indicating an effective mass of 5.4 *m*_e_. With the introduction of Se_O_ and Cu vacancies in P+S-BC_1−*x*_SO, the corresponding effective mass increases to 5.91 *m*_e_ in P+S-BCSO and keeps improving to 7.32 *m*_e_ in P+S-BC_0.95_SO. Considering the simultaneous increase in carrier concentration, this increase in effective mass is closely related to the multiple degenerate hole pockets in the valence band structure of BCSO [49]. As previously reported, there are several sub-valence bands that lie near the valence band maximum (VBM) of BCSO with very close energies, indicating a complex multiple converged valence band character. As the *n* rises, the Fermi level will move down to the valence band and more hole pockets will be occupied, which activates the multiple converged valence bands [49,50], and increases the density of state effective mass m^*^. As a result, the increase in m^*^ in P+S-BC_1−*x*_SO compensates for the reverse increase in *n*, which finally brings about the increase in *S* compared to that of P-BCSO (see Fig. 3C). Based on the measured *σ* and *S*, Fig. 3D presents the calculated *PF* of the samples as a function of temperature. It can be seen that the *PF* of all doped samples has been improved compared to the pure counterpart, among which the sample P+S-BC_0.97_SO (Bi_0.94_Pb_0.06_Cu_0.97_Se_1.05_O_0.95_) shows the highest *PF* of 942 μW m^−1^ K^−2^ at 823 K due to the synergistically improved *σ* and *S*. Figure S7 also shows the repeatability tests of the electrical properties for sample P+S-BC_0.97_SO, which underwent 3 electrical cycle tests and the performances remained basically the same, showing a good cycling stability.

**Fig. 4.**
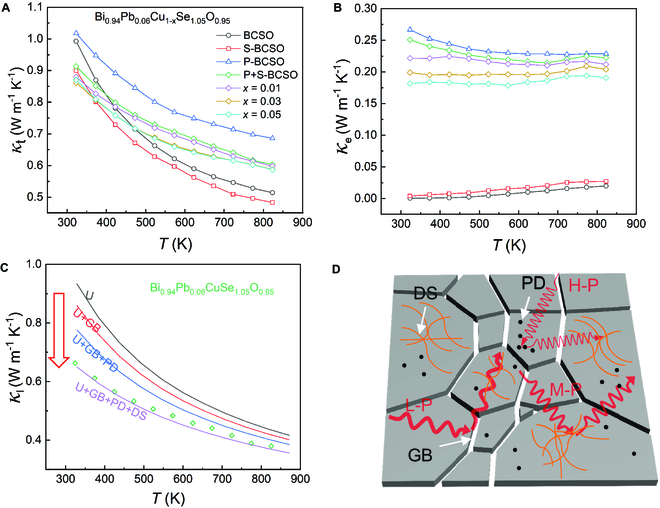
Thermal transport properties. (A and B) Temperature-dependent total thermal conductivity (A) and electronic thermal conductivity (B) of the BiCuSeO, BiCuSe_1.05_O_0.95_, Bi_0.94_Pb_0.06_CuSeO, and Bi_0.94_Pb_0.06_Cu_1−*x*_Se_1.05_O_0.95_ (*x* = 0, 0.01, 0.03, and 0.05) samples. (C) Lattice thermal conductivity modeling based on the Born–von Karman approximation for acoustic phonons and Einstein approximation for optical phonons with different phonon-scattering mechanisms, including the Umklapp-process scattering (U), grain boundary scattering (GB), point defect scattering (PD), and dislocation scattering (DS). For sample P+S-BCSO with dense dislocations, the experimental lattice thermal conductivity (green hollow points) is in good agreement with the simulated values of the purple line by considering the U+GB+PD+DS scattering, indicating that the dense dislocations are indeed responsible for the observed substantial low lattice thermal conductivity. (D) Diagram of the phonon-scattering mechanism in Bi_0.94_Pb_0.06_Cu_1−*x*_Se_1.05_O_0.95_ systems (PD for point defect, DS for dislocation, and GB for grain boundary; L-P, M-P, and H-P indicate low-, mid-, and high-frequency phonons).

Figure 4 shows the results of the thermal transport properties of the samples in the temperature range of 323 to 823 K and illustrates the phonon-scattering mechanism. Figure 4A shows the *κ* of BCSO, S-BCSO, P-BCSO, and P+S-BC_1−*x*_SO (*x* = 0, 0.01, 0.03, and 0.05) samples as a function of temperature. Figure 4B is the corresponding *κ*_e_ calculated according to the Wiedemann−Franz relation, *κ*_e_ *= LσT*. Here, *L* is the Lorentz number (as shown in Fig. S8), which is obtained by fitting the *S* value [49,51]. As shown in Fig. 4A, BCSO inherently has extremely low *κ* due to its special quasi-superlattice layered structure and large Grüneisen parameters caused by Bi^3+^ lone pair electrons and the local vibration of Cu^+^ [52]. When O is substituted by Se, the *κ* of the S-BCSO sample is reduced due to the generation of a large number of dislocations, which complements the scattering of mid-frequency phonons (M-P). Unlike that, when Bi is doped by Pb, although the *κ*_l_ is reduced due to point defect scattering of high-frequency phonons (H-P), *n* is obviously increased, which greatly improves the *κ*_e_ and the *κ* of P-BCSO. Combining the Pb_Bi_ doping and Se_O_ substitution, the *κ*_l_ of Se_O_ and Pb_Bi_ co-doped P+S-BCSO decreases markedly due to additional M-P scattering by the high-density dislocations, so that *κ* is much reduced compared to P-BCSO. With further introduction of Cu vacancies, the *κ*_l_ basically remains unchanged (see Fig. 1E), but the *κ* of P+S-BC_1−*x*_SO (*x* = 0.01, 0.03, and 0.05) continues to decrease, mainly because of the reduction in *κ*_e_ (see Fig. 4B) caused by the reduction of *κ*_e_ (see Fig. 3A).

To quantitatively analyze the function of lattice dislocation in reducing *κ*_l_, *κ*_l_ was modeled according to the Born–von Karman approximation of acoustic phonons and the Einstein approximation of optical phonons [44], with different phonon-scattering mechanisms, including the Umklapp-process scattering (U), grain boundary scattering (GB), point defect scattering (PD), and dislocation scattering (DS). Details of the modeling can refer to the calculation items in the Supporting Information and previous work [42]. As shown in Fig. 4C, the black, red, blue, and purple lines represent the predicted *κ*_l_ considering the contribution of U, U+GB, U+GB+PD, and U+GB+PD+DS, respectively, which demonstrates a descending trend with increasing scattering sources. The green rhombic dots in the figure are the measured experimental values of P+S-BCSO. Obviously, the experimental values are in good agreement with the simulated values of the purple line (U+GB+PD+DS contribution), considering additional dislocation scattering of phonons, indicating that the uniformly distributed dense dislocations in P+S-BCSO are primarily responsible for the observed substantial low *κ*_l_, and their contribution to the *κ*_l_ reduction is estimated to be at least 30% over the whole temperature range. Finally, based on the above discussed scenario, the diagram of the phonon-scattering mechanisms in P+S-BC_1−*x*_SO systems is illustrated in Fig. 4D. Multiple length-scale lattice defects, including nanoscale Se_O_, Pb_Bi_, and V_Cu_ point defects (PD), mesoscale dislocations (DS), and microscale grain boundaries (GB), coexist in the matrix. While PD and GB are preferred for scattering the high-frequency phonons (H-P) and low-frequency phonons (L-P), respectively, DS is particularly effective for mid-frequency phonon (M-P) scattering. Therefore, the combined effect of the multiple scattering mechanisms cooperatively gives rise to an all-scale phonon scattering and results in a minimum *κ*_l_ of 0.38 W m^−1^ K^−1^ at 823 K in the P+S-BC_0.97_SO sample.

**Fig. 5.**
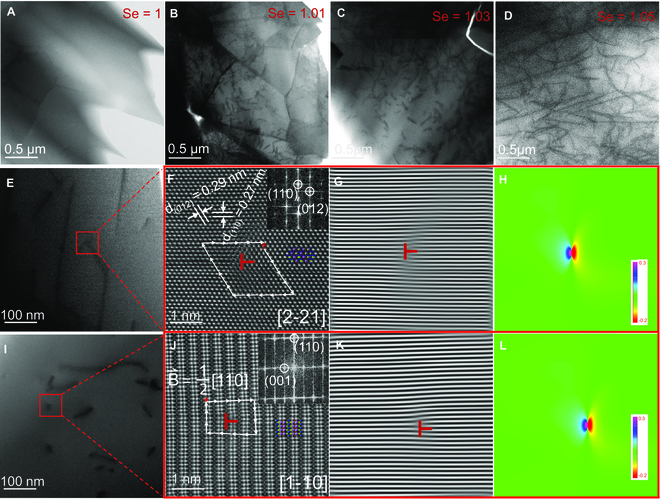
TEM characterizations of the dislocation. (A to D) Low-magnification STEM images of BiCuSe_1+*x*_O_1−*x*_ samples with *x* = 0 (A, Se = 1), 0.01 (B, Se = 1.01), 0.03 (C, Se = 1.03), and 0.05 (D, Se = 1.05), showing that dislocation emerges after Se_O_ self-substitution and its density increases with the increase of Se content. (E) Low-magnification STEM image of sample BiCuSe_1.05_O_0.95_. (F) Enlarged STEM image of the dislocation core in BiCuSe_1.05_O_0.95_ along the [2-21] zone axis. (G and H) The inverse fast Fourier transform (IFFT) images (G), and geometric phase analysis (H) of panel F. (I) Low-magnification STEM image of sample Bi_0.94_Pb_0.06_Cu_0.97_Se_1.05_O_0.95_. (J) Enlarged STEM image of the dislocation in Bi_0.94_Pb_0.06_Cu_0.97_Se_1.05_O_0.95_ along the [1-10] zone axis. The white arrows give a complete Burgers loop of a dislocation, and the estimated Burgers vector (red arrow) for the observed dislocations is $\vec{B}$ = 1/2 [110]. (K and L) The inverse fast Fourier transform (IFFT) images (K) and geometric phase analysis (L) of panel J. Superpositions in panels F and J are the projected atomic structure models of BiCuSeO along the corresponding zone axis.

In order to deeply understand the evolution of these dislocations, we also synthesized Se_O_-single-doped BiCuSeO samples with different Se/O ratios. Figure S9 shows XRD patterns and temperature-dependent thermal transport properties of the BiCuSe_1+*x*_O_1−*x*_ (BCS_1+*x*_O_1−*x*_) samples (0 ≤ *x* ≤ 0.09). It is found in Fig. S9a that when *x* increases above 0.05, the secondary phase of Bi_3_Se_4_ appears in the sample. Meanwhile, the measured *κ* (Fig. S9b) decreases first and then increases with increasing Se content, and shows a minimum value at the Se content of 1.05. By calculating the electronic and lattice components (Fig. S9C and D), we can see that the anomalous variation of *κ* is mainly caused by the *κ*_l_. Figure 5A to D shows the low-magnification STEM images of BCS_1+*x*_O_1−*x*_ samples with a Se content (1+*x*) of 1, 1.01, 1.03, and 1.05, and it can be obviously found that the dislocation in the sample matrix gradually increased with the increase of Se content, which is in accord with the decrease of *κ*_l_ with increasing Se content.

To understand the structure of the dislocation, fine STEM characterizations were also performed on typical BiCuSe_1.05_O_0.95_ (S-BCSO) and Bi_0.94_Pb_0.06_Cu_0.97_Se_1.05_O_0.95_ (P+S-BC_0.97_SO) samples. Figure 5E and I shows the low-magnification STEM images corresponding to S-BCSO and P+S-BC_0.97_SO samples, respectively, where black lines indicating the dislocations can be clearly seen. Figure 5F and J shows the atomic resolution STEM-HAADF images of the demarcated red areas in the 2 samples along the [2-21] and [1-10] zone axes, and the insets show the fast Fourier transform (FFT) maps of the corresponding positions. The projected atomic structures along the corresponding zone axis are overlapped in the figures, which are in agreement with the atomic row in the HAADF image [28]. The projected Burgers vector of dislocations can be determined by plotting the Burgers loop. The white arrows in Fig. 5J give a complete Burgers loop of a dislocation, and the Burgers vector (red arrow in the figure) is estimated to be $\vec{B}$=1/2 [110] when observed along the [1-10] zone axis, which corresponds to the Burgers vector of dislocations common to tetragonal phase structures [53,54]. Figure 5G and K shows the inverse FFT (IFFT) maps by using the reflection 110, from which we can clearly see a semi-atomic plane insertion, confirming the existence of edge dislocations. In order to resolve the strain close to the dislocation region, experimental images were processed by geometric phase analysis and the ε*_xx_* strain distribution is shown in Fig. 5H and L. The purple and red colors represent the maximum compressive and tensile strains, respectively. It can be seen that the dislocation cores exhibit obvious strain concentration, which indicates that the lattice strain caused by dislocations due to Se_O_ self-substitution in BiCuSeO may be the main reason for the extremely low *κ*_l_.

## Conclusion

In summary, by turning our sights to the thermoelectric material itself and activating the function of intrinsic defects, the present work reports the successful introduction of high-density in-grain dislocations by Se_O_ self-substitution in BiCuSeO oxide thermoelectrics, and achieves plain optimization of the thermoelectric properties with less use of external Pb doping. Through self-substitution of Se at the O site, the lattice is distorted to a certain extent, which promotes the formation of in-grain lattice dislocations. The dislocation density gradually increases with the increase of Se_O_ concentration and reaches a high density of ~3.0 × 10^14^ m^−2^ in the Bi_0.94_Pb_0.06_Cu_0.97_Se_1.05_O_0.95_ sample. These dense dislocation defects complement the weakness in the mid-frequency phonon scattering and lead to a full-scale phonon scattering with a minimum lattice thermal conductivity of 0.38 W m^−1^ K^−1^ at 823 K. Meanwhile, Pb_Bi_ doping and Cu vacancy markedly improve the electrical conductivity while maintaining a competitively high Seebeck coefficient, thus contributing to the much-enhanced power factor with a highest value of 942 μW m^−1^ K^−2^ in Bi_0.94_Pb_0.06_Cu_0 .97_Se_1.05_O_0.95_. As a result, due to the solidarity of the electrical and thermal properties, the final thermoelectric figure of merit is substantially improved compared to the pristine BiCuSeO and a highest *zT* value of 1.32 is obtained at 823 K in the Bi_0.94_Pb_0.06_Cu_0.97_Se_1.05_O_0.95_ sample. The much-improved *zT* performance is obtained by tailoring the intrinsic defects with only external Pb dopant, which embodies the concept of “plain optimization” and would promote the recycling of corresponding thermoelectric materials. In addition, the high-density dislocation defects in BiCuSeO by Se_O_ self-substitution also provide a good inspiration for the design and construction of dislocations in other oxide systems.

## Materials and Methods

### Material preparation

BiCuSeO, BiCuSe_1.05_O_0.95_, Bi_0.94_Pb_0.06_CuSeO, and Bi_0.94_Pb_0.06_Cu_1−*x*_Se_1.05_O_0.95_ (*x* = 0, 0.01, 0.03, and 0.05) polycrystalline bulks were prepared by solid-state reaction from Bi_2_O_3_ (99.99%), PbO (99.99%), Cu (99.99%), Bi (99.99%), and Se (99.99%) raw materials, followed by a rapid hot press process to densify the powders. Firstly, the raw materials were mixed in stoichiometric proportions and sealed in a vacuum quartz tube, which was heated at 573 K for 9 h and 973 K for 6 h to ensure that the chemical reaction was complete. After natural cooling to room temperature, the product was ground to a fine powder. Subsequently, the round block material was sintered in a graphite mold at 873 K for 30 min under vacuum and a uniaxial pressure of 70 MPa using a rapid hot-press equipment. The round block material obtained by hot pressing was cut to the specified size with a diamond wire cutter to test the thermoelectric properties.

### Sample characterization

XRD (DX−2700B) was used to reveal the phase composition of the BiCuSeO, BiCuSe_1.05_O_0.95_, Bi_0.94_Pb_0.06_CuSeO, and Bi_0.94_Pb_0.06_Cu_1−*x*_Se_1.05_O_0.95_ (*x* = 0, 0.01, 0.03, and 0.05) samples with Cu K_α_ radiation in a 2θ range of 10° to 80° with a step of 0.04°. Transmission electron microscope (TEM, JEOL-F200), aberration-corrected transmission electron microscope (TEM, JEOL-NEOARM 200F), EDS, and BSE imaging (ZEISS Crossbeam 550 FIB-SEM) were used to reveal the phase composition of the samples. Transmission electron microscope samples for TEM characterization were prepared using grinding, polishing, and argon ion thinning in the liquid nitrogen stage with the Ion Beam Milling System (GATAN PIPS II 695). It should be noted that the use of lightweight argon ion in the liquid nitrogen cold stage for sample thinning can effectively minimize the damage to the sample and avoid ion bombardment-induced dislocations. The IFFT image was obtained by processing the HRTEM image in the Gatan Digital Micrograph (GMS-3) software.

### Thermoelectric performance evaluation

The electrical conductivity and Seebeck coefficient were measured in He atmosphere from 323 to 823 K in a commercial system (LSR-3, Linseis) with a sample size of about 10 mm × 3 mm × 3 mm. The thermal diffusivity (*D*) was measured by the laser flash method (LFA 457, Netzsch) in the same temperature range with a sample size of about ϕ 12.7 mm × 1.8 mm; the specific heat (*C*p) was calculated according to the Dulong–Petit law and the relative bulk density (*ρ*) was measured by the Archimedes method. The total thermal conductivity *κ* was calculated with the relation *κ =ρ·D·C*p*.* The carrier concentration and mobility were measured by the Van der Pauw method using the DC field Hall effect measurement option installed on the standard Model 8404 (HMS 8400 series, Lake Shore).

## Data Availability

The data are available from the authors upon a reasonable request.

## Supplementary Materials

Supplementary Materials Fig. S1. TEM images of the BCSO, P-BCSO, S-BCSO, and P+S-BC_0.97_SO samples. Fig. S2. Comparison of highest *zT* with literature reported values. Fig. S3. STEM-HADDF and elemental mapping images of the P+S-BC0.97SO sample. Fig. S4. The ΔK-K^2^C plots for the S-BCSO and P+S-BCSO samples. Fig. S5. Carrier concentration and mobility, Pisarenko curves. Fig. S6. BSE and elemental mapping images of the BCSO and S-BCSO samples. Fig. S7 Repeatability tests of electrical properties of the P+S-BC_0.97_SO sample. Fig. S8. Calculated Lorentz number. Fig. S9. XRD pattern, electrical thermal conductivity, lattice thermal conductivity, and total thermal conductivity of the BCS_1+*x*_O_1−*x*_ samples (0 ≤ *x* ≤ 0.09). Table S1. Parameters used for the lattice thermal conductivity modeling. Calculation Items for Lattice Thermal Conductivity.
