# Supplementary material for: Realizing Plain Optimization of the Thermoelectric Properties in BiCuSeO Oxide via Self-Substitution-Induced Lattice Dislocations
Source: Research (Wash D C). 2023 Apr 18;6:0123. doi: 10.34133/research.0123 (PMC10243199; doi:10.34133/research.0123)
Supplement: Supplementary Materials — Fig. S1. TEM images of the BCSO, P-BCSO, S-BCSO, and P+S-BC0.97SO samples. Fig. S2. Comparison of highest zT with literature reported values. Fig. S3. STEM-HADDF and elemental mapping images of the P+S-BC0.97SO sample. Fig. S4. The ΔK-K2C plots for the S-BCSO and P+S-BCSO samples. Fig. S5. Carrier concentration and mobility, Pisarenko curves. Fig. S6. BSE and elemental mapping images of the BCSO and S-BCSO samples. Fig. S7 Repeatability tests of electrical properties of the P+S-BC0.97SO sample. Fig. S8. Calculated Lorentz number. Fig. S9. XRD pattern, electrical thermal conductivity, lattice thermal conductivity, and total thermal conductivity of the BCS1+xO1−x samples (0 ≤ x ≤ 0.09). Table S1. Parameters used for the lattice thermal conductivity modeling. Calculation Items for Lattice Thermal Conductivity. [file research.0123.f1.docx]

**Supplementary Materials**

**Realizing Plain Optimization of the Thermoelectric Properties in BiCuSeO Oxide via Self-substitution-induced Lattice Dislocations**

Rui Xu^1,2^, Zhiwei Chen^3^, Qizhu Li^1^, Xiaoyu Yang^1^, Han Wan^1^, Mengruizhe Kong^4^, Wei Bai^4^, Nengyuan Zhu^1^, Ruohan Wang^1^, Jiming Song^1^*, Zhou Li^1,2^*, Chong Xiao^2,4^, Binghui Ge^1,2^*

*^1^Information Materials and Intelligent Sensing Laboratory of Anhui Province, Key Laboratory of Structure and Functional Regulation of Hybrid Materials of Ministry of Education, Institutes of Physical Science and Information Technology and School of Materials Science and Engineering, Anhui University, Hefei, 230601, China*

*^2^Institute of Energy, Hefei Comprehensive National Science Center, Hefei, 230031, China*

*^3^School of Materials Science and Engineering, Tongji University, Shanghai, 201804, China*

*^4^Hefei National Laboratory for Physical Sciences at the Microscale, CAS Center for Excellence in Nanoscience, University of Science and Technology of China, Hefei, 230026, China*

**Contents of Supporting Information**

**1. Supplementary Figures and Tables**

**Figure S1.** TEM images of BCSO, P-BCSO, S-BCSO, P+S-BC0_.97_SO samples.

**Figure S2.** Comparison of highest *zT* with literature reported values.

**Figure S3.** STEM-HADDF and elemental mapping images of P+S-BC_0.97_SO sample.

**Figure S4.** The △K-K^2^C plots for S-BCSO and P+S-BCSO samples.

**Figure S5.** Carrier concentration and mobility, Pisarenko curves.

**Figure S6.** BSE and elemental mapping images of BCSO and S-BCSO samples.

**Figure S7.** Repeatability tests of electrical properties of P+S-BC_0.97_SO sample.

**Figure S8.** Calculated Lorentz number.

**Figure S9.** XRD pattern, electrical thermal conductivity, lattice thermal conductivity and total thermal conductivity of BCS_1+x_O_1-x_ samples (0≤x≤0.09).

**Table S1.** Parameters used for the lattice thermal conductivity modeling.

**2. Supplementary Calculation Items for Lattice Thermal Conductivity**

**3. Supplementary References**

**1. Supplementary Figures and Tables**.


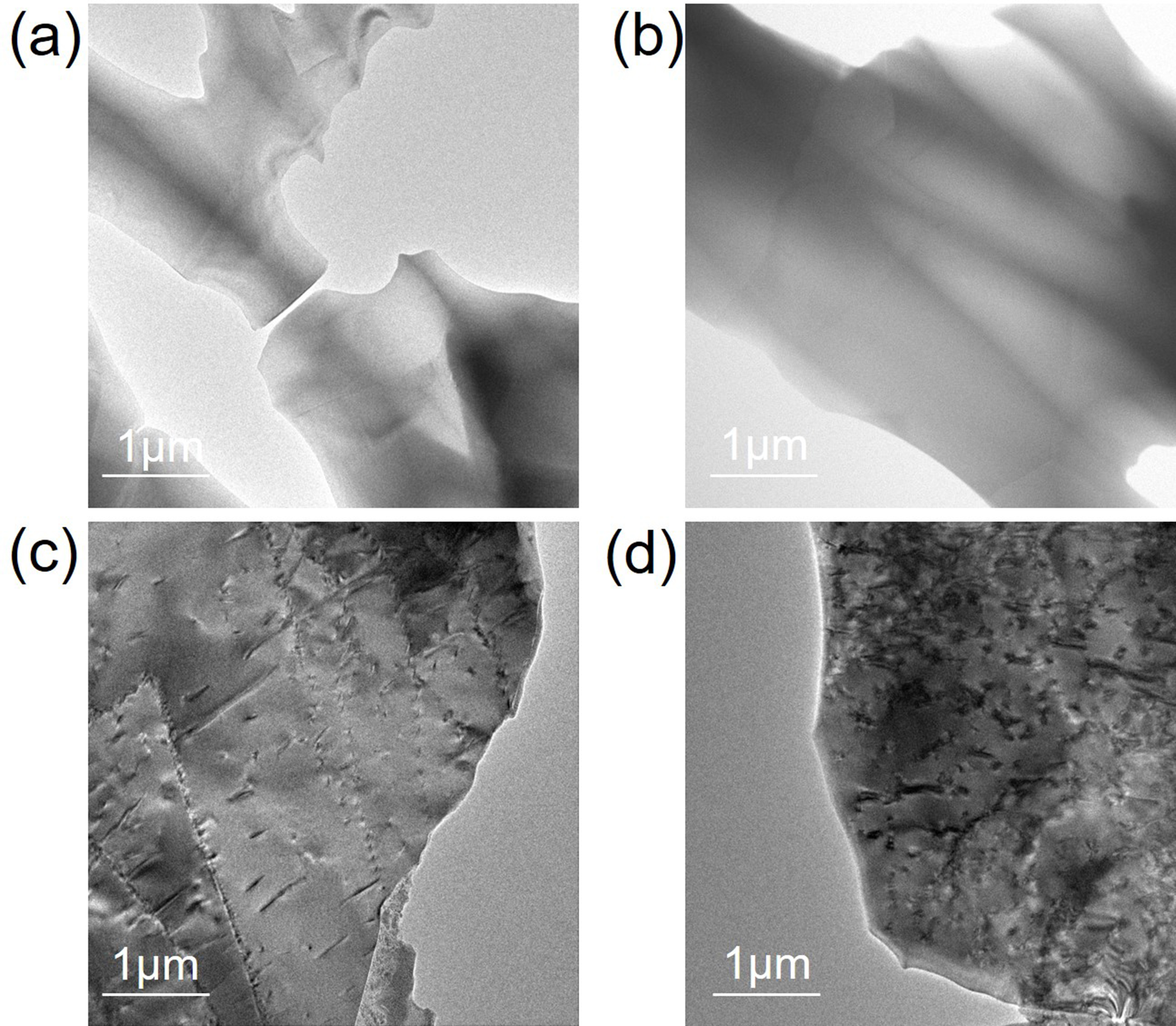


**Figure S1.** (a) TEM image of BiCuSeO (BCSO). (b) TEM image of Bi_0.94_Pb_0.06_CuSeO (P-BCSO). (c) TEM image of BiCuSe_1.05_O_0.95_ (S-BCSO). (d) TEM image of Bi_0.94_Pb_0.06_CuSe_1.05_O_0.95_ (P+S-BCSO).


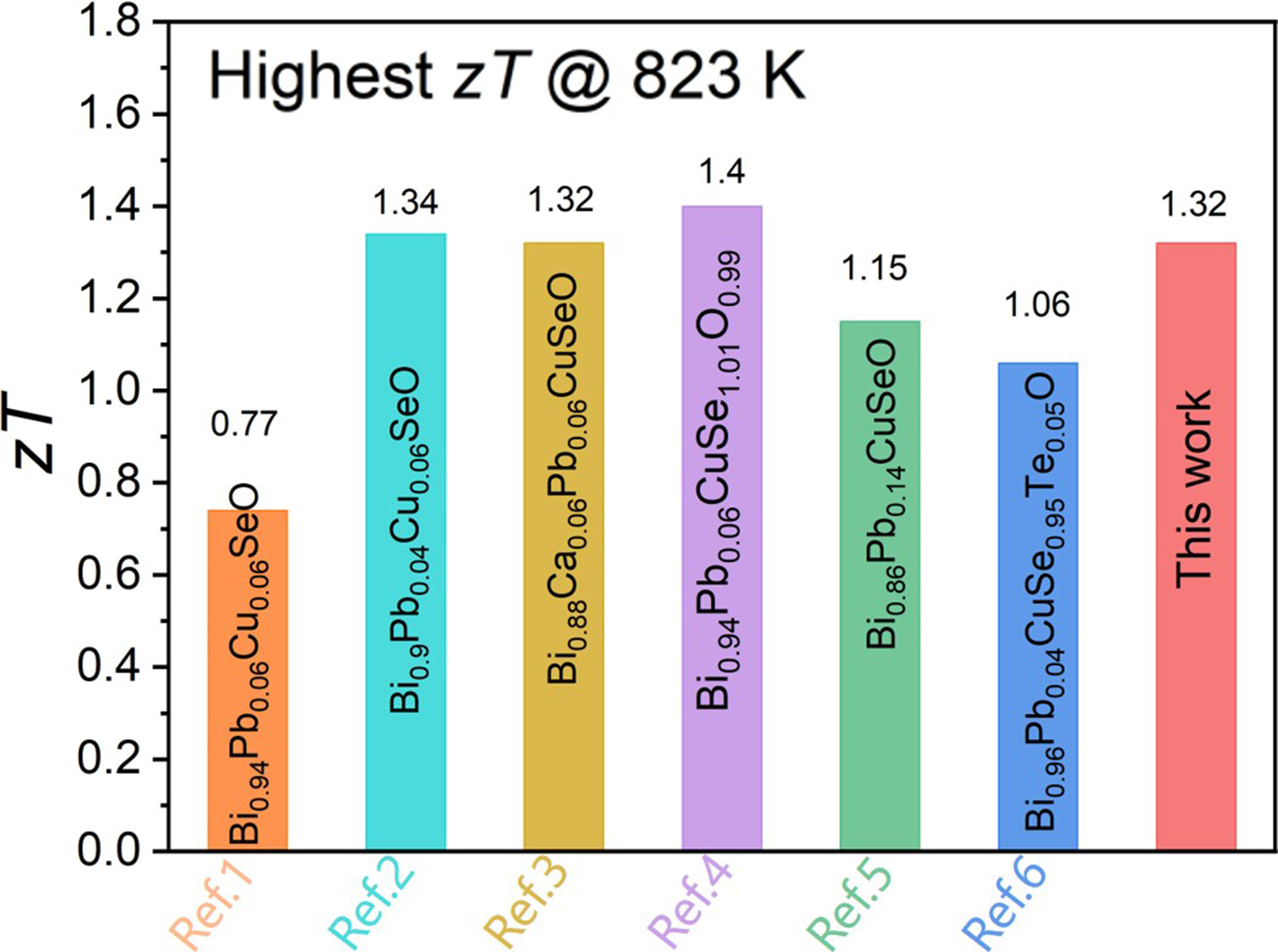


**Figure S2.** Comparison of the highest *zT* value in present work with other reported BiCuSeO-based compounds at 823 K [1−6].


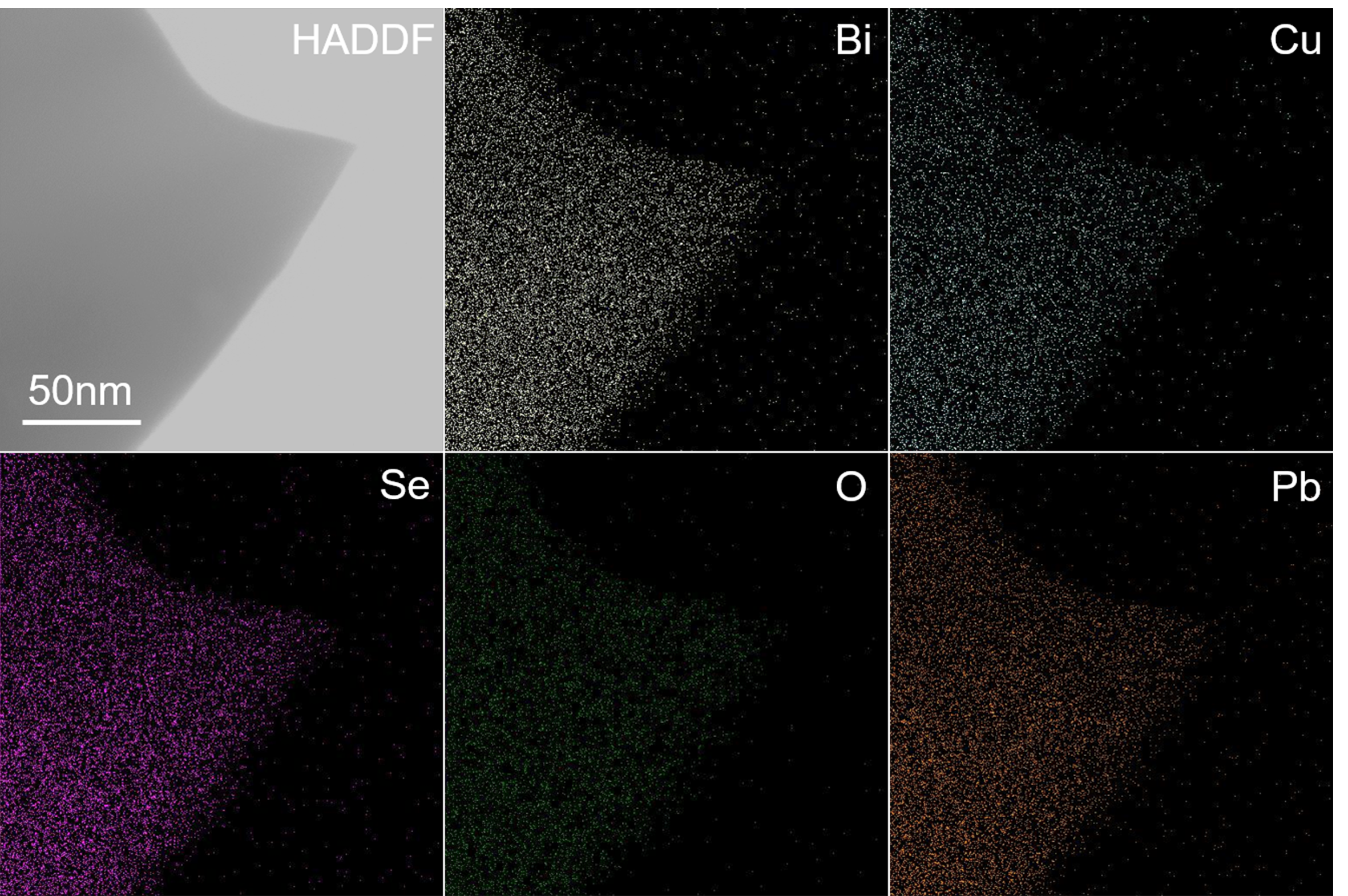


**Figure S3**. STEM-HADDF and nanoscale EDS elemental mapping images of sample Bi_0.94_Pb_0.06_Cu_0.97_Se_1.05_O_0.95_, showing that all elements in the region are uniformly distributed, without enrichment or precipitation.


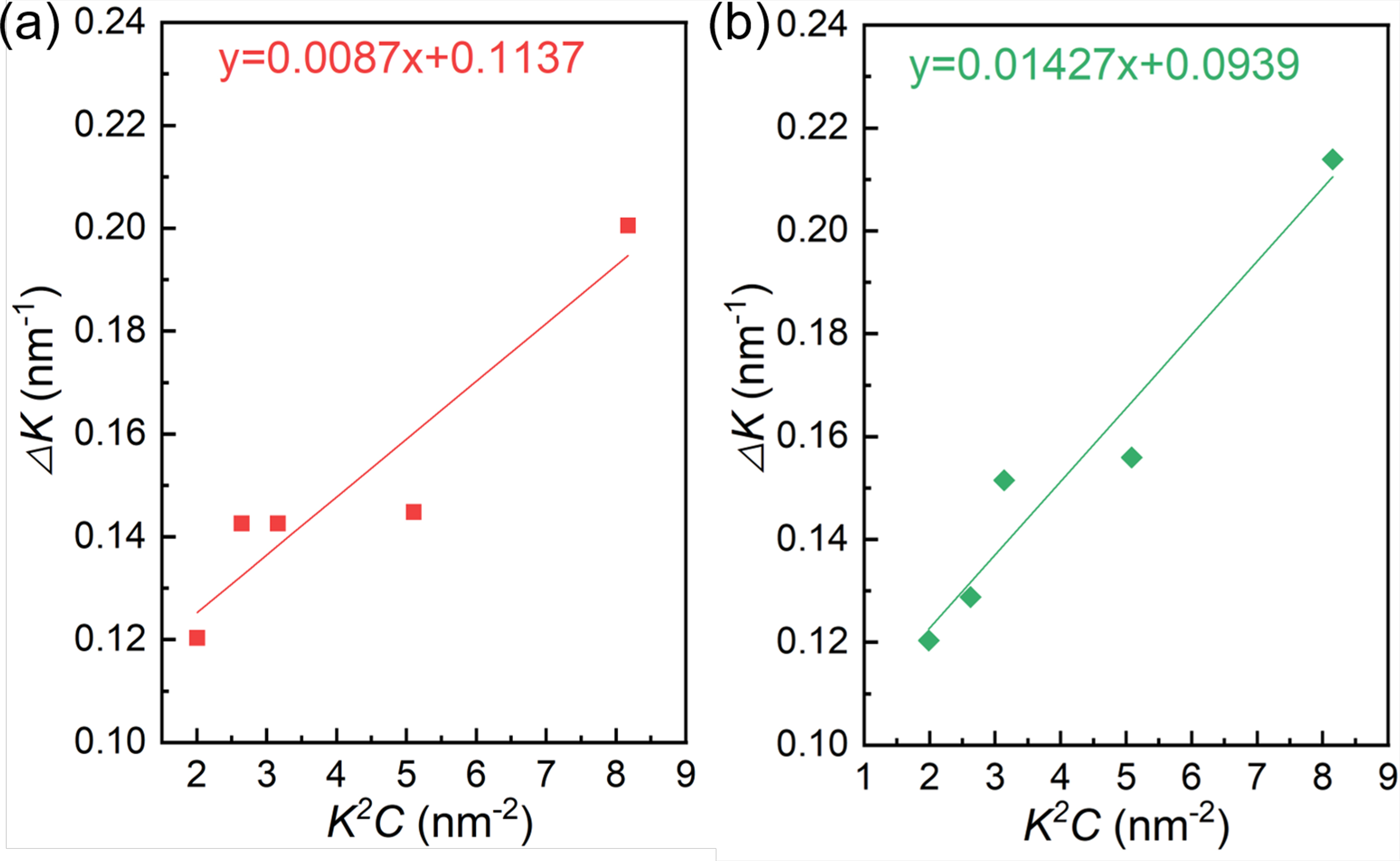


**Figure S4.** The △K-K^2^C plots based on the modified Williamson-Hall analysis of the XRD diffraction peak widths for S-BCSO (a) and P+S-BCSO (b) samples, in which the slope of the plots reveal the dislocation density.


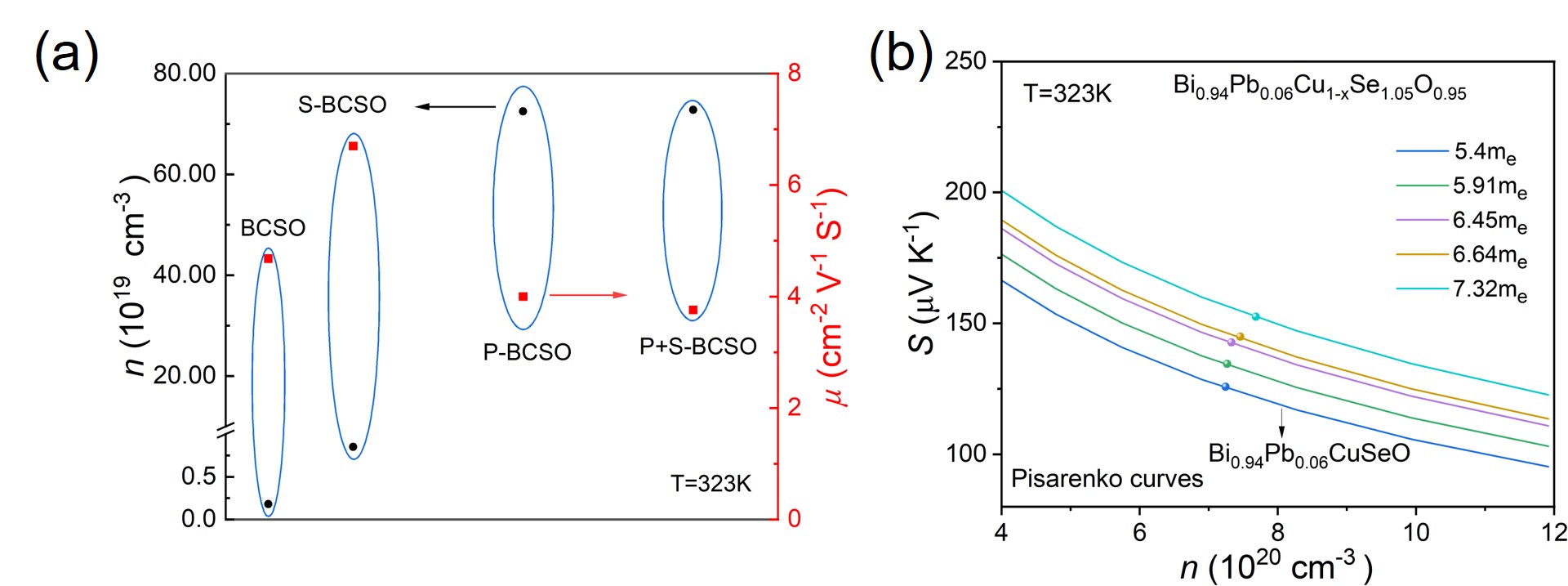


**Figure S5.** (a) Carrier concentration and mobility of BCSO, S-BCSO, P-BCSO, and P+S-BCSO samples. (b) Pisarenko curves of P-BC_1-x_SO.


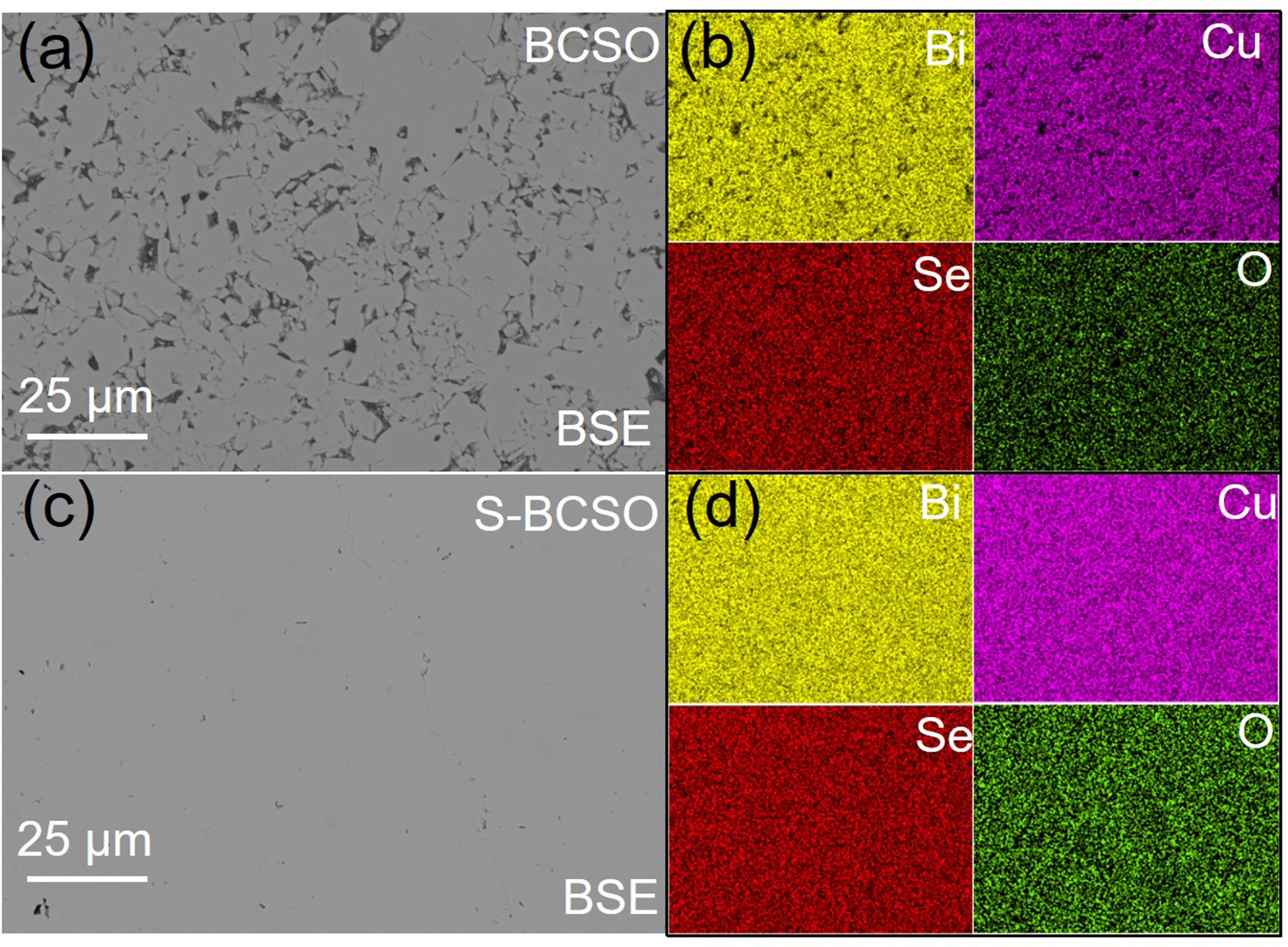


**Figure S6.** (a, b) BSE image of BCSO (a) and the corresponding elemental mapping results with Bi, Cu, Se, and O elements (b). (c, d) BSE image of S-BCSO (c) and the corresponding elemental mapping results with Bi, Cu, Se, and O elements (d).


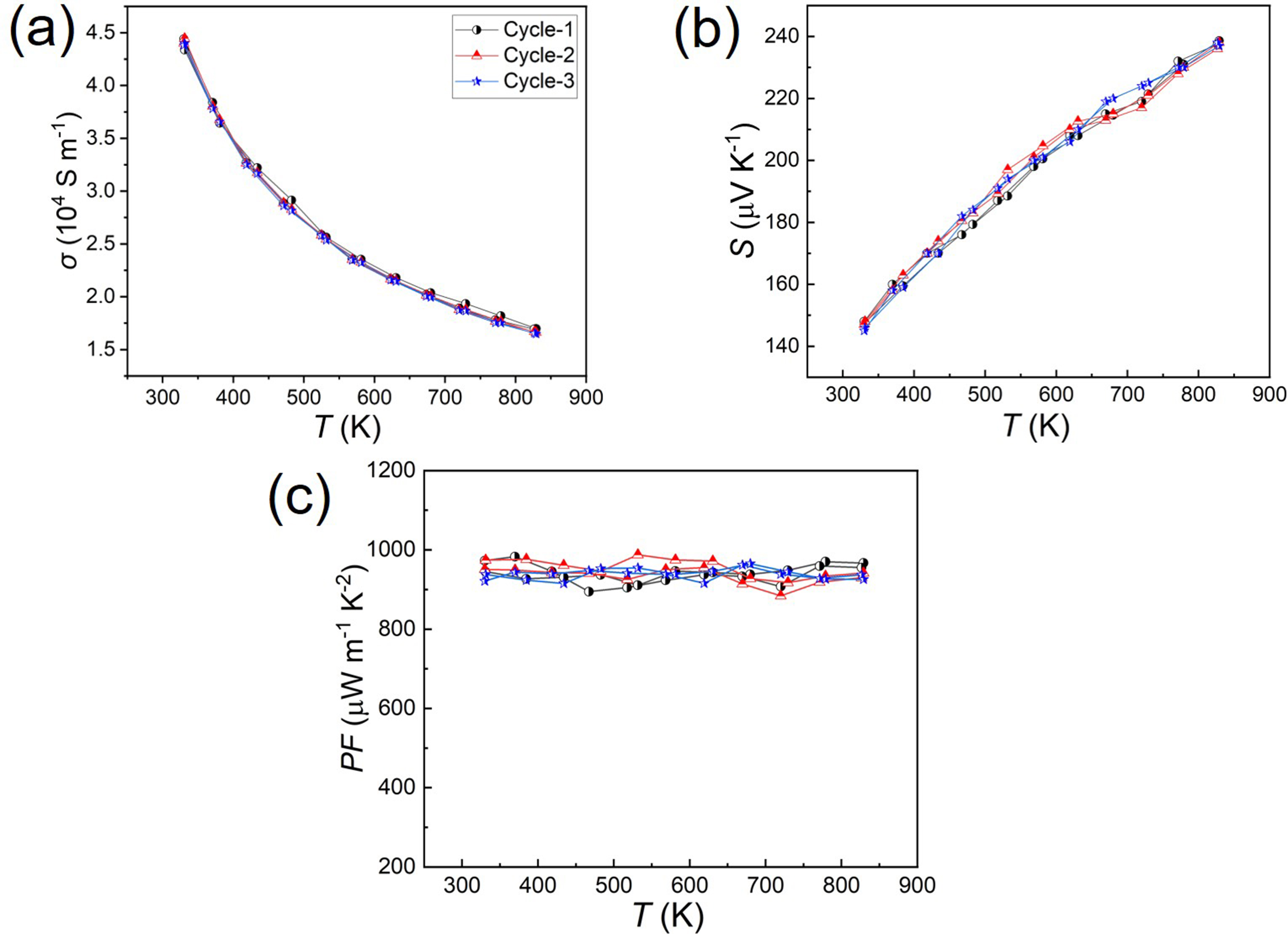


**Figure S7.** (a−c) Repeatability tests of electrical properties of sample P+S-BC_0.97_SO, including the electrical conductivity (a), Seebeck coefficient (b), and power factor (c), showing a good cycling stability.


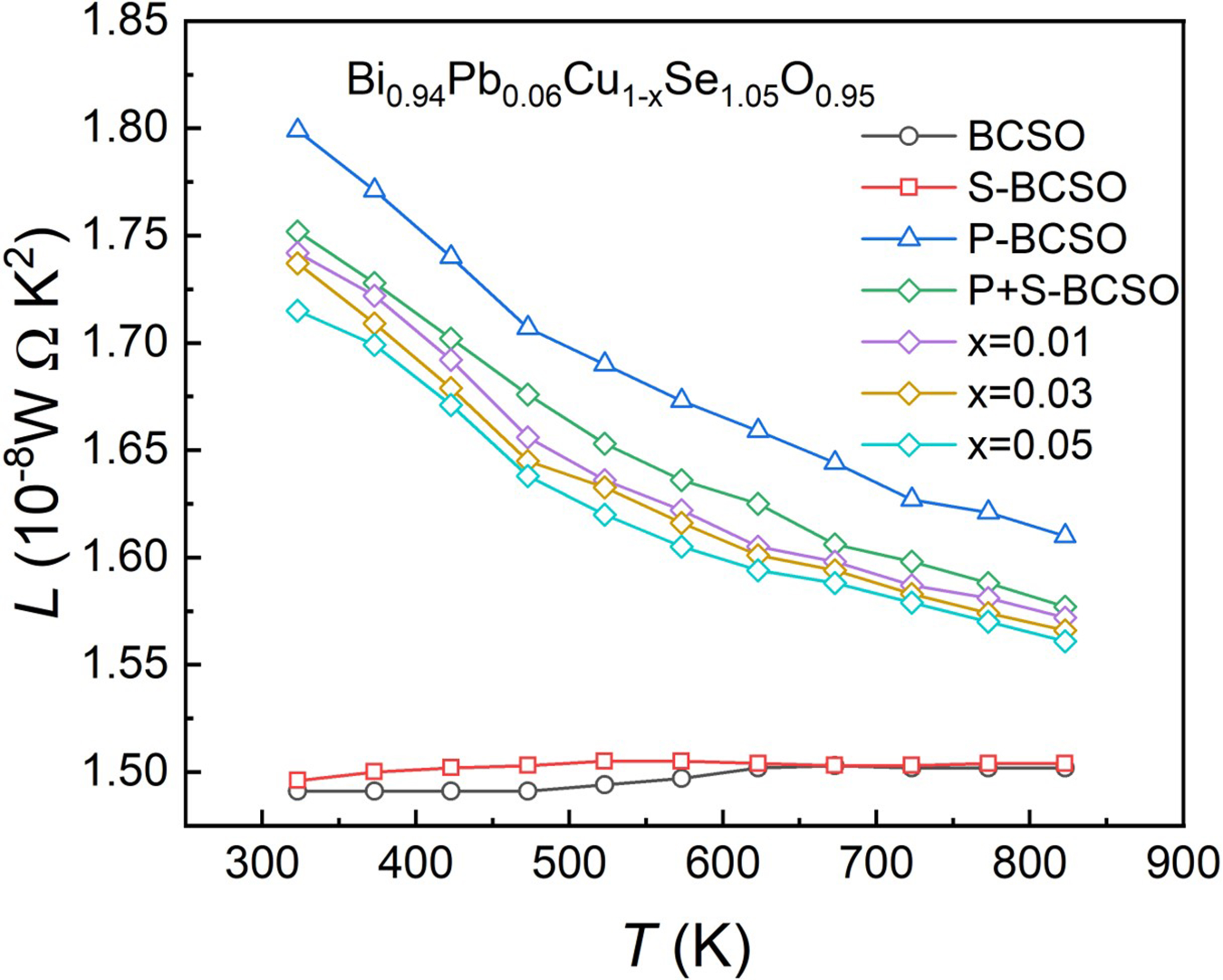


**Figure S8.** Temperature-dependent Lorenz number of all samples.


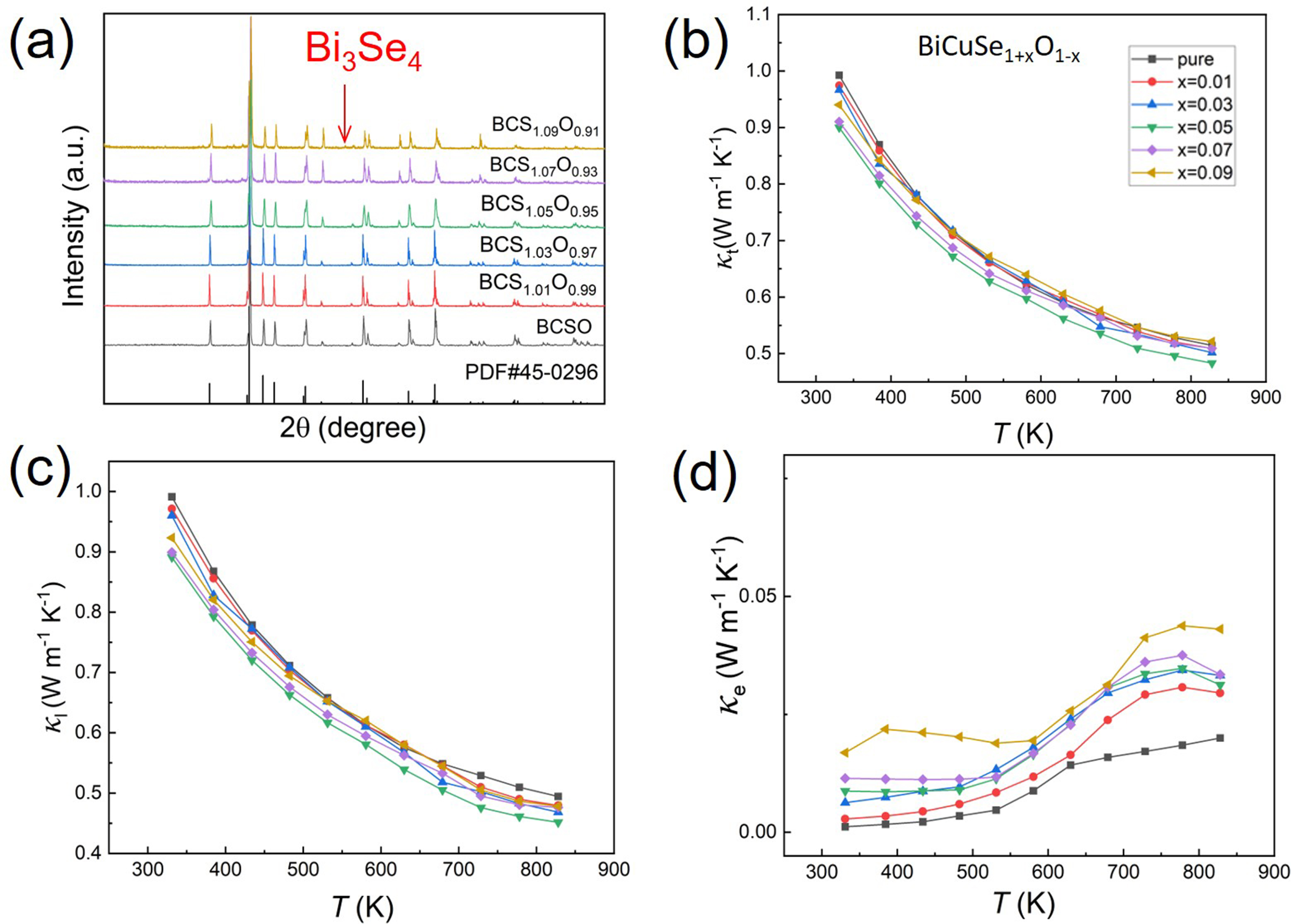


**Figure S9.** (a) XRD patterns of the BCS_1+x_O_1-x_(0≤x≤0.09) samples. (b−d) The temperature-dependent total thermal conductivity (b), lattice thermal conductivity (c), and electronic thermal conductivity (d) of BCS_1+x_O_1-x_ samples (0≤x≤0.09).

**Table S1.** Parameters used for lattice thermal conductivity modeling.

| Parameters | Description | Values |
| --- | --- | --- |
| $\overline{M}$ | Average atomic mass for P+S-BCSO | M_P+S-BCSO_ = (2×6.023×10^-23^) kg |
| $\overline{V}$ | Average atomic volume of P+S-BCSO | $\frac{{a_{i}}^{2}b_{i}}{8}$ m^3^ |
| $\upsilon$ | Average sound speed | 2107 m s^-1^ |
| $\upsilon_{L}$ | Longitudinal sound speed | 3290 m s^-1^ |
| $\upsilon_{T}$ | Transverse sound speed | 1900 m s^-1^ |
| $\gamma$ | Gruneisen parameter | 2.5 [7,8] |
| $x_{i}$ | Impurities concentration in solid solutions | $x_{Pb}=0.06$ |
| $M_{i}$ | Atomic mass of impurities | $M_{Pb}=207.2$ g mol^-1^ |
| $M$ | Atomic mass of matrix | $M_{Bi}=208.98$ g mol^-1^ |
| $a_{i},b_{i}$ | Lattice parameters for P+S-BCSO | $a_{i}=3.9404$Å,$b_{i}=8.9779$Å |
| $a,b$ | Lattice parameters for BCSO | $a=3.927$Å,$b=8.92$Å |
| $N_{D}$ | Dislocation density of P+S-BCSO | $3.0\times10^{14}\text{m}^{\text{-2}}$ |
| $B_{D}$ | Burgers vector | $5\times10^{-10}m$ |
| $r$ | Poisson’s ratio | 0.25 |
| *k*_c_ | Cutoff wave vector | 6p^2^/($\overline{V}$*N_pri_* )^1/3^ |
| $\omega_{a}$ | Acoustic cutoff frequency | $(2/\pi)\upsilon k_{c}$ |
| *N_pri_* | Number of atoms in the protocell | 8 |
| *d* | Grain size | 3×10^-6^ m |

**2. Supplementary Calculation Items for Lattice Thermal Conductivity**

The lattice thermal conductivity and phonon relaxation time can be determined by the following formulas [9]:

$\kappa_{L}=\frac{1}{3}\int_{0}^{\omega_{a}} C_{V}\left( \omega\right)\upsilon_{g}^{2}\tau d\omega$ (1)

$\frac{1}{\tau}=\gamma^{2}\varepsilon^{2}+\left( \frac{1}{\overline{M}} \right)^{2}\Delta M^{2}$ (2)

$\omega_{a}$is the cutoff frequency, $\omega$ is the frequency where $\omega=\frac{2}{\pi}\upsilon k_{c}sin(\frac{\pi}{2}\frac{k}{k_{c}})$, *C_V_* is the specific heat capacity, υ*_g_* is the group velocity where $\upsilon_{g}=\upsilon cos(\frac{\pi}{2}\frac{k}{k_{c}})$, *τ* is the relaxation time of the phonon, γ is the Gruneisen constant, *ε* is the strain fluctuation, $\overline{M}$ is the average atomic mass, and *ΔM* is the mass fluctuation.

The equations for strain fluctuations and mass fluctuations can be determined by the following formulas:

$\varepsilon^{2}=A+B\varepsilon_{PD}^{2}+C\varepsilon_{DS}^{2}++D\varepsilon_{GB}^{2}$ (3)

$\Delta M^{2}=E\left( \Delta M_{PD} \right)^{2}+G\left( \Delta M_{DS} \right)^{2}$ (4)

where *A* is the U-process scattering, *B*, *C* and *D* are the collected physical constant for lattice strain fluctuations due to point defects, dislocations and grain boundaries, respectively. *E* and *G* denote the collected physical constant for mass fluctuations due to point defects and dislocations. *PD*, *DS* and *GB* refer to the subscript for the effect due to point defects, dislocations and grain boundaries.

The pre-factor *A* of the U-process can be written as:

$A=\frac{2}{\left( 6\pi^{2} \right)^{\frac{1}{3}}}\frac{k_{B}\overline{V}^{\frac{1}{3}}\omega^{2}T}{\overline{M}\upsilon_{g}\upsilon_{p}^{2}}$ (5)

where 𝑘_B_ is the Boltzmann constant, $\overline{V}$ is the average atomic volume, 𝑇 is the absolute temperature, 𝑣_p_ is the phase velocity where $\upsilon_{p}=(\frac{2}{\pi}\upsilon k_{c}\sin\left( \frac{\pi}{2}\frac{k}{k_{c}} \right))/k$.

The pre-factor *B* due to point defects can be written as:

$B=\frac{\overline{V}\omega^{4}}{4\pi\upsilon_{g}\upsilon_{p}^{2}}8\left( 1+Q \right)^{2}$ (6)

where *Q*=3.2 which means the anharmonicity of the nearest elastic constants is excluded.

According to the formula of Klemens[10], the interatomic elasticity caused by the absence of isolated or uniformly distributed points is constant. The variation of number can be expressed by its strain field. The lattice strain due to point defects can be calculated by the following equation:

$\varepsilon_{PD}^{2}=\sum ix_{i}\left( \frac{R_{i}-\overline{R}}{\overline{R}} \right)^{2}=\sum ix_{i}\left( \frac{11+r}{31-r} \right)^{2}\left( \frac{a_{i}-\overline{a}}{\overline{a}} \right)^{2}$ (7)

where 𝑥*_i_* is the concentration of solute *i*, 𝑅*_i_* is the ionic radius of solute *i*, $\overline{R}$ is the average ionic radius, 𝑟 is the Poisson’s ratio (calculated from Leont’ev formula [11]), 𝑎*_i_* the lattice constant of solute *i*, $\overline{a}$ is the average lattice constant.

The pre-factor *C* due to edge dislocation can be written as:

$C=\frac{2}{3}\frac{32\pi^{2}\left( r^{2}-1 \right)^{2}}{3-2r+3r^{2}}\omega\ln\frac{\frac{1}{2}\sqrt{N_{D}}}{b}$ (8)

where *b* is the Burgers vector, *N_D_* is the dislocation density.

The lattice strain due to dislocations can be calculated by the following equation:

$\varepsilon_{DS}^{2}=\frac{3-2r+3r^{2}}{32\pi^{2}\left( r^{2}-1 \right)^{2}}\frac{b^{2}}{\left( \frac{1}{2}\sqrt{N_{D}} \right)^{2}}\ln\frac{\frac{1}{2}\sqrt{N_{D}}}{b}$ (9)

The pre-factor *D* is:

$D=\frac{0.12}{d}\upsilon_{g}$ (10)

And the strain due to grain boundaries is:

$\varepsilon_{GB}^{2}=\frac{\phi^{2}}{4}N_{GB}$ (11)

In this work, *φ*^2^*N_GB_*~1. The pre-factor *D* due to quality rise and fall caused by point defects can be written as:

$E=\frac{\overline{V}\omega^{4}}{4\pi\upsilon_{g}\upsilon_{p}^{2}}$ (12)

The mass rise and fall caused by point defects can be expressed by the following equation:

$\Delta M_{PD}^{2}=\sum ix_{i}\left( M_{i}-\overline{M} \right)^{2}$ (13)

The pre-factor *D* due to quality rise and fall caused by dislocations can be written as:

$G=\frac{\overline{V}^{\frac{4}{3}}\omega^{3}}{\upsilon_{g}\upsilon_{p}}$ (14)

The mass fluctuations caused by dislocations can be expressed by the following equation:

$\Delta M_{DS}^{2}=N_{D}\left( 0-\overline{M} \right)^{2}$ (15)

It should be noted that the following assumptions were used in the calculations:

1. Acoustic phonons and optical phonons are considered separately, with acoustic phonons having sinusoidal dispersion and optical phonons being a series of Einstein models;

2. Each scattering mechanism acts mainly on acoustic phonons, and optical phonons are treated with minimum relaxation time;

3. An isotropic assumption is made.

**3. Supplementary References**

[1] Y.-X. Chen, K.-D. Shi, F. Li, X. Xu, Z.-H. Ge, J. He, "Highly enhanced thermoelectric performance in BiCuSeO ceramics realized by Pb doping and introducing Cu deficiencies," *Journal of the American Ceramic Society*, vol. 102, no. 10, pp. 5989-5996, 2019.

[2] H. Zhu, Z. Li, C. Zhao, X. Li, J. Yang, C. Xiao, Y. Xie, "Efficient interlayer charge release for high-performance layered thermoelectrics," *National Science Review*, vol.8, no.2, pp nwaa085, 2021.

[3] Y. Liu, L.-D. Zhao, Y. Zhu, Y. Liu, F. Li, M. Yu, D.-B. Liu, W. Xu, Y.-H. Lin, C.-W. Nan, "Synergistically Optimizing Electrical and Thermal Transport Properties of BiCuSeO via a Dual-Doping Approach," *Advanced Energy Materials*, vol. 6, no. 9, article 1502423, 2016.

[4] Y. Gu, X. L. Shi, L. Pan, W. D. Liu, Q. Sun, X. Tang, L. Z. Kou, Q. F. Liu, Y. F. Wang, Z. G. Chen, "Rational Electronic and Structural Designs Advance BiCuSeO Thermoelectrics," *Advanced Functional Materials*, vol. 31, no. 28, article 2101289, 2021.

[5] G. K. Ren, S. Wang, Z. Zhou, X. Li, J. Yang, W. Zhang, Y. H. Lin, J. Yang, C. W. Nan, "Complex electronic structure and compositing effect in high performance thermoelectric BiCuSeO," *Nature Communications,* vol. 10, no. 1, article 2814, 2019.

[6] G.-K. Ren, S.-Y. Wang, Y.-C. Zhu, K. J. Ventura, X. Tan, W. Xu, Y.-H. Lin, J. Yang, C.-W. Nan, "Enhancing thermoelectric performance in hierarchically structured BiCuSeO by increasing bond covalency and weakening carrier–phonon coupling," *Energy & Environmental Science*, vol. 10, no. 7, pp. 1590-1599, 2017.

[7] J. Ding, B. Xu, Y. Lin, C. Nan, W. Liu, "Lattice vibration modes of the layered material BiCuSeO and first principles study of its thermoelectric properties," *New Journal of Physics*, vol. 17, no. 8, article 083012, 2015.

[8] H. Shao, X. Tan, G. Q. Liu, J. Jiang, H. Jiang, "A first-principles study on the phonon transport in layered BiCuOSe," *Scientific Reports*, vol. 6, article 21035, 2016.

[9] P. G. Klemens, "Thermal Conductivity and Lattice Vibrational Modes," *Academic Press*, vol. 7, pp. 1-98, 1958.

[10] P. G. Klemens, "The Scattering of Low-Frequency Lattice Waves by Static Imperfections," *Proceedings of the Physical Society*, vol. A68, pp. 1113, 1958.

[11] S. K. Srivastava, "Relationship between elastic constants and thermal expansivity for ionic solids," *Physica B*, vol. 287, no. 1-2, pp. 396-399, 2007.
